# Surgical resection combined with TACE for primary clear cell carcinoma of the liver: a case report and literature review

**DOI:** 10.3389/fsurg.2026.1949319

**Published:** 2026-10-19

**Authors:** Liying Qian, Kangze Wu, Yukun Ying, Xin Zhu, Zhihong Shen

**Affiliations:** 1 Department of Hepatobiliary Surgery, Shaoxing People’s Hospital, Shaoxing, China; 2 School of Medicine, Shaoxing University, Shaoxing, Zhejiang, China; 3 Shaoxing Key Laboratory of Minimally Invasive Abdominal Surgery and Precise Treatment of Tumor, Shaoxing People's Hospital, Shaoxing, Zhejiang, China

**Keywords:** HCC, histopathology, laparoscopic liver resection, PCCCL, TACE

## Abstract

**Background:**

Primary clear cell carcinoma of the liver (PCCCL) is an uncommon hepatocellular carcinoma (HCC) subtype that resembles extrahepatic clear cell tumors, posing diagnostic challenges.

**Case presentation:**

A 74-year-old woman with hepatitis B–related cirrhosis (Child-Pugh class A) presented with acute abdominal pain and vomiting. Imaging revealed a hypervascular hepatic lesion with intralesional fat, while serum AFP was normal and CA125 mildly elevated. Laparoscopic partial hepatectomy was performed. Histology confirmed PCCCL, and immunohistochemistry supported hepatic origin while excluding metastatic renal carcinoma. The patient subsequently received two sessions of postoperative TACE as an individualized treatment and remained free of recurrence or metastasis during surveillance from 2022 to 2025.

**Conclusion:**

PCCCL is rare and difficult to distinguish from conventional HCC or metastatic clear cell carcinoma. Histopathology with immunohistochemistry is essential for diagnosis. Surgical resection is the mainstay of treatment. Although this patient remained recurrence-free after postoperative TACE, the role of TACE cannot be determined based on a single case, and studies with larger sample sizes are still needed.

## 1 Introduction

Hepatocellular carcinoma (HCC) remains a major global health burden, ranking as the sixth most common malignancy and the third leading cause of cancer-related mortality worldwide. Its pathogenesis is multifactorial, with well-established risk factors including aflatoxin exposure, chronic alcohol consumption, hepatitis B and C virus infections, obesity, and related metabolic disorders (1). Among its histological variants, primary clear cell carcinoma of the liver (PCCCL) represents an uncommon subtype with a remarkably low incidence (2–4) Notably, the morphological features of PCCCL closely resemble those of clear cell carcinomas originating from extrahepatic organs such as the kidney, adrenal gland, ovary, pancreas, and lung (5, 6), creating substantial diagnostic challenges. Accurate distinction between PCCCL and metastatic clear cell carcinoma is clinically crucial, as the therapeutic strategies and prognostic implications differ significantly. Here, we report a case of a small PCCCL that was preoperatively evaluated using contrast-enhanced ultrasonography, computed tomography (CT), and magnetic resonance imaging (MRI), with the final diagnosis confirmed by histopathological examination.

## 2 Case presentation

A 74-year-old Asian woman presented with a 3-day history of abdominal pain accompanied by vomiting. During this period, she experienced recurrent episodes of abdominal pain radiating to the back with a tightening sensation, followed by vomiting of dark, ’soy sauce–colored’ fluid, occurring 5–6 times with a total volume of approximately 200 mL. She denied fever, abdominal distension, jaundice, or other associated symptoms. She had a history of chronic hepatitis B virus (HBV) infection and compensated liver cirrhosis. She had not received regular antiviral therapy, and the serum HBV DNA level was 9.07 × 10^6. She had previously undergone splenectomy for hypersplenism secondary to cirrhosis. There was no history of variceal bleeding, hepatic encephalopathy, or other episodes of hepatic decompensation. On physical examination, mild tenderness was noted in the upper abdomen, with no additional positive findings.

To further evaluate her condition, contrast-enhanced abdominal CT revealed a poorly defined, nodular, low-density lesion measuring approximately 6 mm in diameter located near the diaphragmatic surface of the left hepatic lobe, demonstrating mild enhancement (Figure 1). Subsequent contrast-enhanced MRI identified an abnormal nodular lesion in segment 4 of the liver, measuring approximately 10 × 7 mm, characterized by hypointensity on T1-weighted images, slight hyperintensity on T2-weighted images, high signal on DWI, and signal drop on opposed-phase imaging. The lesion showed ill-defined margins and progressive, persistent enhancement after contrast administration, suggestive of a hypervascular lesion with intralesional fat (Figure 2). A malignant hepatic tumor was therefore suspected.

**Figure 1:**
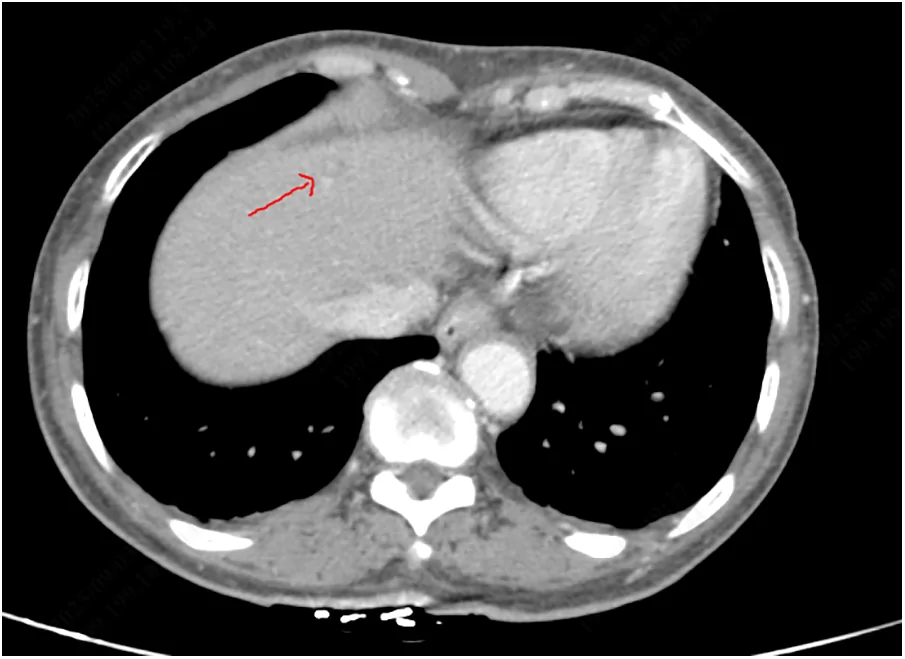
Contrast-enhanced CT of the patient. A poorly defined, nodular hypodense lesion measuring approximately 6 mm was observed near the diaphragmatic surface of the left hepatic lobe, showing mild enhancement after contrast administration.

**Figure 2:**
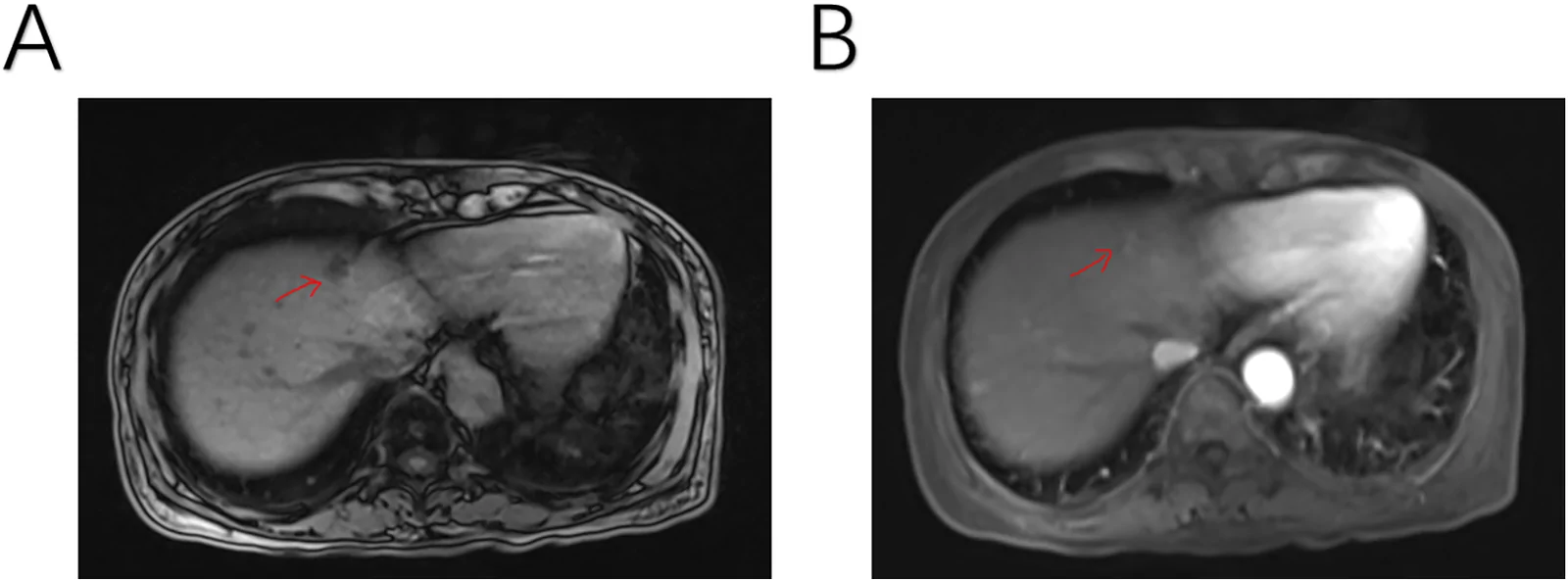
Contrast-enhanced MRI of the patient. (**A**) An abnormal nodular lesion approximately 10 × 7 mm in size was detected in segment 4 of the liver. The lesion was hypointense on T1-weighted images, slightly hyperintense on T2-weighted images, hyperintense on DWI, and demonstrated signal drop on opposed-phase imaging. (**B**) The lesion showed ill-defined margins as well as progressive and persistent enhancement following contrast administration, suggestive of a hypervascular lesion containing intralesional fat.

Laboratory investigations showed a white blood cell count of 5.71 × 10^9/L and hemoglobin of 113 g/L. Serum albumin was 32.6 g/L, total bilirubin was 23.3 μmol/L, and prothrombin time was 14.8 s. A small amount of peritoneal fluid was observed but was not considered clinically significant cirrhotic ascites, and hepatic encephalopathy was absent. The Child-Pugh score was 6 (class A). Tumor markers showed normal AFP and CA19–9 levels and mildly elevated CA125 (47.17 U/mL).

Further assessment was performed with contrast-enhanced ultrasound, which demonstrated a hyperechoic lesion in the left hepatic lobe measuring 15 × 14 mm with irregular margins and internal punctate blood flow. Following intravenous administration of contrast agent, the lesion exhibited marked arterial-phase hyperenhancement at 25 s, exceeding that of the surrounding liver parenchyma, followed by early washout at 41 s, consistent with imaging features of HCC (Figure 3).

**Figure 3:**
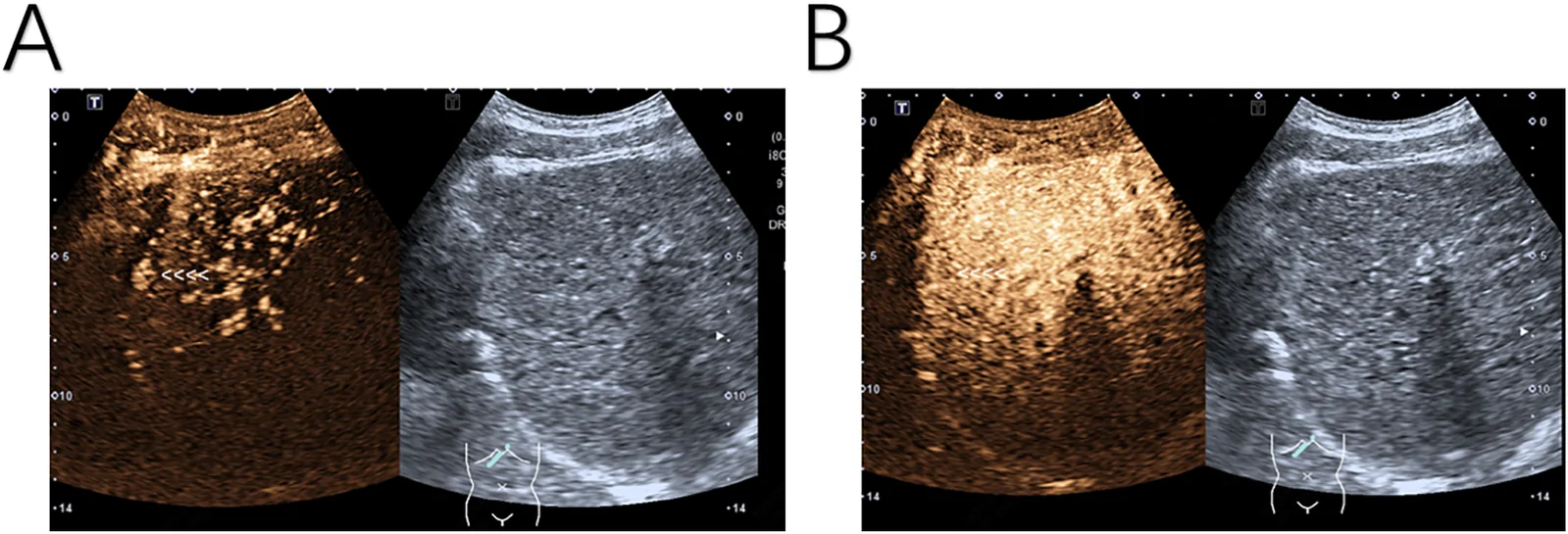
Contrast-enhanced ultrasound of the patient. (**A**) A hyperechoic lesion measuring 15 × 14 mm was identified in the left hepatic lobe, presenting with irregular morphology and internal punctate blood flow. After intravenous contrast injection, the lesion displayed marked hyperenhancement at 25 s in the arterial phase, which was stronger than that of the surrounding liver parenchyma. (**B**) Subsequent washout was observed at 41 s, with the lesion appearing hypoechoic compared with the adjacent liver tissue.

Following multidisciplinary discussion, the patient underwent laparoscopic non-anatomical partial hepatectomy with intraoperative ultrasonographic localization. The operative record described incision of the liver capsule approximately 3 cm from the tumor, followed by complete excision. Indocyanine green fluorescence imaging was not used. Histopathological examination confirmed negative resection margins (R0).

Initial frozen-section examination suggested an atypical proliferative hepatic lesion with focal infiltrative growth. Subsequent examination of permanent paraffin-embedded sections stained with hematoxylin and eosin (H&E) showed tumor cells with abundant clear cytoplasm, with clear cells accounting for more than 90% of the tumor. The final pathological diagnosis was PCCCL. Representative low- and high-power H&E images are presented in Figures 4A,B. Resection margins were negative, and no major vascular invasion, microvascular invasion (MVI M0), satellite nodules, or pseudocapsule were identified. Immunohistochemistry showed AFP (−), CEA (−), P53 (partial +), CK7 (−), CK19 (−), CgA (−), Ki-67 (5%), Hepatocyte (+), MLH1 (+), MSH2 (+), MSH6 (+), PMS2 (+), CD10 (+), CK20 (−), Vimentin (−), CD56 (−), S-100 (−), HMB-45 (−), CD34 (−), Hep-1 (+), Synaptophysin (−), and RCC (−). These findings supported hepatocellular differentiation. Additional periodic acid–Schiff (PAS) and PAS with diastase (PAS-D) staining was performed on paraffin-embedded tumor tissue. PAS staining showed cytoplasmic positivity, which was markedly reduced after diastase digestion, supporting glycogen accumulation in the clear tumor cells (Figures 4C,D).

**Figure 4:**
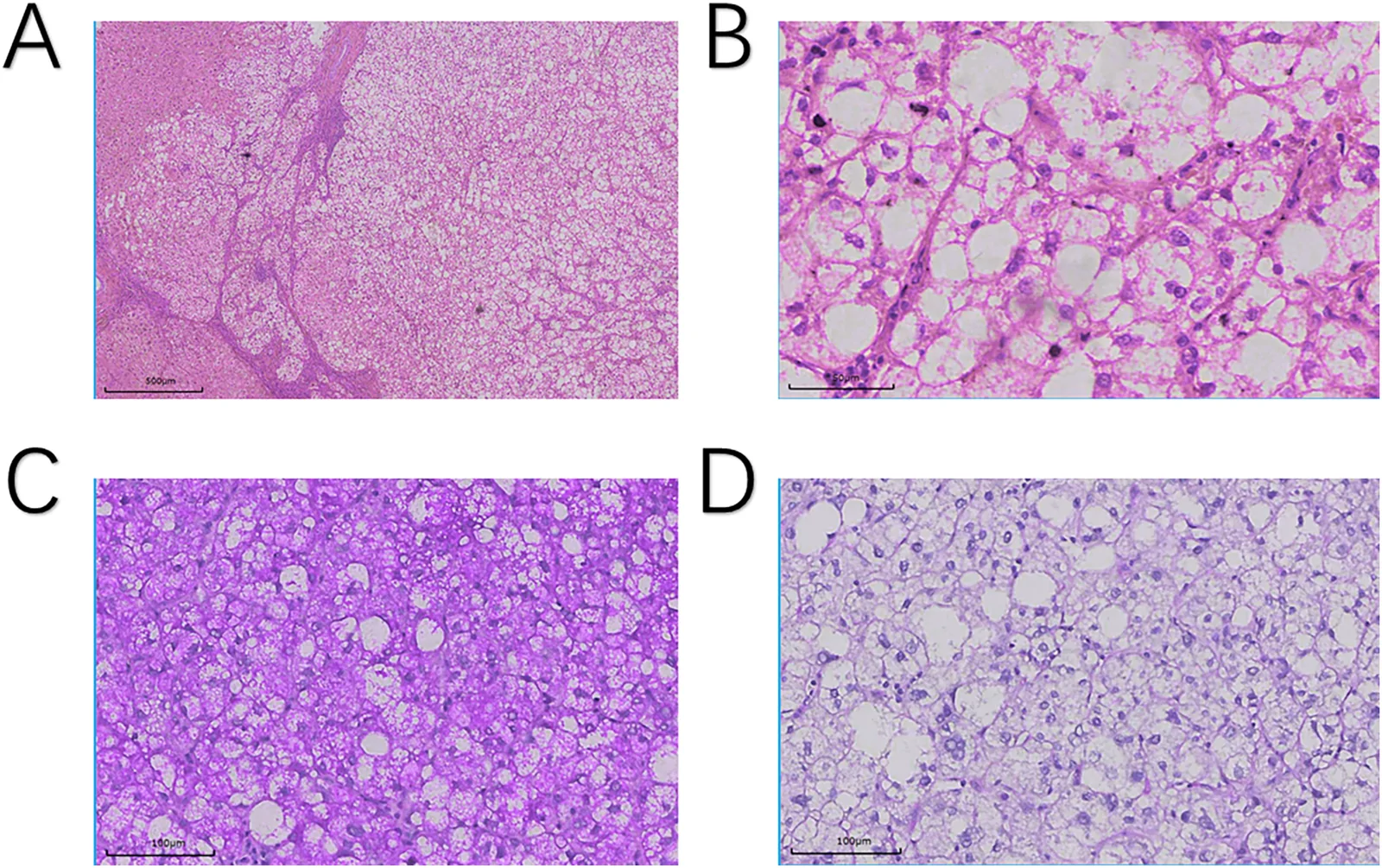
Histopathological examination of permanent paraffin-embedded tumor sections. **(A)** Low-power H&E image showing the tumor architecture ( × 40; scale bar, 500 μm). **(B)** High-power H&E image showing cells with abundant clear cytoplasm (  × 400; scale bar, 50 μm). **(C)** PAS staining showing cytoplasmic positivity (  × 200; scale bar, 100 μm). **(D)** PAS-D staining showing markedly reduced cytoplasmic staining after diastase digestion (  × 200; scale bar, 100 μm). Clear cells accounted for more than 90% of the tumor on pathological review.

Postoperatively, the patient received two sessions of transarterial chemoembolization (TACE) as individualized adjuvant therapy, with no treatment-related adverse events observed. In addition, long-term antiviral therapy for HBV infection was initiated after surgery. From 2022 to 2025, the patient underwent regular postoperative surveillance. Serial clinical examinations revealed no significant abnormalities, and follow-up assessments showed no evidence of recurrence, metastasis, or death. At the last follow-up in 2025, the patient remained alive and free of recurrence or metastasis (Table 1).

**Table 1:** Timeline.

Time	Clinical event
Preoperative period	Abdominal pain and vomiting; CT, MRI, and contrast-enhanced ultrasonography identified a very small hypervascular hepatic lesion with intralesional fat.
Surgery	Intraoperative ultrasonography-guided laparoscopic non-anatomical partial hepatectomy; negative margins and R0 resection
Postoperative period	Histopathology and immunohistochemistry confirmed PCCCL; two sessions of postoperative TACE were administered without adverse events; long-term antiviral therapy was maintained.
2022–2025	The patient remained alive and free of recurrence or metastasis

## 3 Discussion

PCCCL is a histological subtype of HCC that typically occurs in men aged 50–75 years with a background of cirrhosis, although female patients are not uncommon (4, 7). Our case involved a 74-year-old woman with cirrhosis, consistent with the demographic spectrum reported in the literature. Selected pathological, imaging, surgical, and population-based studies are summarized in Table 2 and discussed below in relation to this case.

**Table 2:** Selected studies included in the focused narrative literature review.

Study	Design/population	Principal findings	Relevance and limitations
Murakata et al., 2000	Comparative pathology; 10 clear cell HCCs and 10 renal clear cell carcinomas	Hepatocellular immunostaining assisted lineage differentiation.	Supports an integrated panel; small sample and no replacement for morphology.
Ji et al., 2010	Retrospective surgical cohort; 64 patients; >50% clear-cell criterion	No significant survival advantage from postoperative calcium folinate/tegafur chemotherapy.	Systemic chemotherapy findings cannot be equated with evidence about TACE.
Kida et al., 2012	Single case report	Hand-assisted laparoscopic hepatectomy for PCCCL.	Establishes prior use of a laparoscopic approach; no comparative efficacy estimate.
Wang et al., 2014	Retrospective CT series; 19 patients	CT appearances overlapped with HCC; enhancement and capsule findings varied.	Imaging may raise suspicion but does not establish the histological subtype.
Chen et al., 2016	Retrospective hepatectomy cohort; 64 PCCCL patients; >30% criterion	Size, portal vein tumor thrombosis, and clear-cell proportion were associated with prognosis.	Historical subtype definition and surgical selection limit generalizability.
Wen et al., 2021	Population-based registry study; 223 patients	Clinical characteristics and surgery were associated with survival.	Registry associations do not establish causality or an adjuvant TACE benefit.

The clinical manifestations of PCCCL are generally nonspecific, including abdominal pain, jaundice, unexplained fever, anorexia, weakness, weight loss, or hepatic decompensation; some patients may even remain asymptomatic until the tumor is incidentally discovered during physical examination (8). This nonspecific presentation complicates the initial diagnosis. In our case, the patient presented with an acute abdomen characterized by abdominal pain and vomiting, which lacked specificity and could have diverted clinical attention to other causes.

MRI and contrast-enhanced CT are the most frequently used diagnostic modalities. Prior studies have shown that PCCCL shares imaging features with conventional HCC, such as early arterial enhancement and rapid washout in the portal venous phase. The presence of a pseudocapsule is considered a distinguishing feature that helps differentiate PCCCL from other hepatic tumors (9). MRI can also identify intralesional lipid content, which manifests as signal drop on opposed-phase imaging, similar to clear cell renal cell carcinoma (10–12) However, not all PCCCLs display such typical features. In our patient, CT revealed a mildly enhancing hypodense lesion, mimicking an atypical hemangioma. MRI showed signal drop on opposed-phase imaging, but this did not allow reliable distinction from metastatic clear cell carcinoma of renal origin. Consequently, contrast-enhanced ultrasound was performed to complement the diagnostic workup and refine surgical decision-making.

Published CT/MRI studies describe substantial overlap between PCCCL and conventional HCC (3, 9). Wang et al. reported variable CT appearances in 19 patients (9). Hu et al. described multimodal imaging of PCCCL in 2024, including CT, MRI, and PET/CT, followed by pathological confirmation after liver transplantation (13). That report and the present case illustrate the role of pathological assessment when imaging suggests malignancy but does not establish the subtype.

PCCCL has also been reported to frequently present as tumors <1 cm in diameter, which is uncommon for HCC and may assist in differentiation (7, 14, 15). The lesion in our patient measured approximately 1 cm, consistent with these findings. Notably, the tumor size measurements differed slightly among CT, MRI, and ultrasonography. We considered these discrepancies more likely to reflect differences in imaging techniques and measurement methods rather than true rapid tumor growth over a short period

With regard to tumor markers, prior studies suggest that PCCCL does not differ significantly from conventional HCC in the rate of AFP positivity (3, 4). In our case, however, AFP was negative, whereas CA125 was elevated, further complicating the diagnostic process.

Histopathology remains the gold standard for diagnosis. When clear cell morphology is encountered, immunohistochemical findings should be interpreted in conjunction with the histopathological features to establish hepatocellular differentiation and exclude metastatic clear cell carcinoma. In the present case, immunohistochemical staining was positive for Hepatocyte and Hep-1 and negative for RCC. Together with the remaining immunophenotypic findings, these results supported a hepatic rather than renal origin of the tumor. PET/CT was not performed in this patient. Although PET/CT may provide complementary information for excluding an extrahepatic primary clear cell malignancy, its sensitivity may be limited for very small lesions. Therefore, the final diagnosis in this case was established primarily on the basis of histopathological findings and comprehensive immunohistochemical evaluation.

Murakata et al. demonstrated the value of hepatocellular markers in distinguishing clear cell HCC from renal clear cell carcinoma (6). Morphology and immunohistochemistry provide complementary information. Hepatocyte and Hep-1 positivity, interpreted with the remaining panel and clinical findings, supported hepatocellular differentiation (6). PAS staining with diastase comparison can demonstrate cytoplasmic glycogen; this characterizes the clear-cell appearance but does not by itself establish the tumor's tissue of origin. Thus, these stains complement morphological and immunophenotypic assessment.

Historical definitions have varied: Ji et al. used a clear-cell proportion exceeding 50%, whereas Chen et al. used a threshold exceeding 30% (16, 17). Currently, there is no consensus regarding the proportion of clear cells required for the diagnosis of PCCCL. In the present case, clear cells accounted for more than 90% of the tumor on pathological review of the permanent sections

Surgical resection remains the mainstay of treatment for PCCCL. Due to its higher tendency for encapsulation, complete excision of encapsulated tumors offers a survival advantage (18). Patients who do not undergo surgery have a shorter median overall survival (OS) compared with those treated surgically (7). Similar to renal cell carcinoma, PCCCL does not appear to respond favorably to conventional radiotherapy (7). Our patient underwent laparoscopic partial hepatectomy followed by two sessions of TACE. Although the benefit of postoperative TACE in HCC remains controversial, some studies suggest that it may reduce short-term recurrence (19). A study by Wang et al. involving 280 patients demonstrated that postoperative adjuvant TACE could significantly reduce tumor recurrence and improve recurrence-free survival as well as overall survival in patients with intermediate- or high-risk HBV-related HCC (19). Nevertheless, these findings cannot be directly extrapolated to patients with PCCCL, and the 3-year recurrence-free survival observed in our patient should not be interpreted as evidence of the efficacy of adjuvant TACE. Alternative explanations for this favorable outcome include the small tumor size, achievement of R0 resection, absence of vascular invasion and satellite nodules, and potentially favorable tumor biology. Accordingly, in this case, postoperative TACE should be regarded as an individualized treatment decision rather than a recommended therapeutic strategy for PCCCL. Further studies involving larger patient cohorts are warranted to clarify the potential efficacy and clinical value of TACE as an adjuvant treatment for PCCCL.

The biological behavior of PCCCL is generally regarded as slow-growing, less aggressive, and of relatively low malignancy. However, prognosis remains debated. One study reported poor outcomes, with a 5-year survival rate of 24.3% and a median OS of 21 months (7), whereas another series of 11 patients demonstrated a favorable prognosis with a 5-year OS of 58.5% (20). This patient remained recurrence-free during documented surveillance from 2022 to 2025, but longer follow-up remains.

This study has several limitations. First, as a single-case report, it cannot establish the efficacy of any postoperative treatment. Second, the potential effect of postoperative TACE cannot be distinguished from the influence of the tumor's inherently favorable clinicopathological features. Third, PET/CT was not performed, which limited further evaluation for an occult extrahepatic primary malignancy. Finally, although the 3-year recurrence-free follow-up provides valuable longitudinal information, longer-term surveillance is warranted given the incompletely characterized natural history of PCCCL.

## 4 Conclusion

In conclusion, because the imaging features of PCCCL overlap with those of conventional hepatocellular carcinoma and metastatic clear cell tumors, definitive diagnosis requires careful histopathological and immunohistochemical evaluation. Complete surgical resection remains the mainstay of treatment for localized disease. Although our patient received postoperative transarterial chemoembolization (TACE) and remained recurrence-free during the 3-year follow-up period, the specific contribution of TACE to this favorable outcome cannot be determined. Future multicenter studies are needed to further define optimal postoperative management strategies and identify prognostic factors in PCCCL.

## Data Availability

The raw data supporting the conclusions of this article will be made available by the authors, without undue reservation.
